# Contrast‐Enhanced Voiding Urosonography for Diagnosis of Ectopic Ureters in Five Female Dogs

**DOI:** 10.1111/vru.70240

**Published:** 2026-09-23

**Authors:** Georgia Trikoupi, Lysimachos Papazoglou, Paraskevi Papadopoulou

**Affiliations:** ^1^ Companion Animal Clinic Faculty of Veterinary Medicine Aristotle University of Thessaloniki Thessaloniki Greece

**Keywords:** canine, contrast‐enhanced voiding urosonography (CE‐VUS), ectopic ureter, ultrasonography, urinary incontinence, urosonography

## Abstract

The aim of the study was to describe the implementation of contrast‐enhanced voiding urosonography (CE‐VUS), a new, alternative, radiation‐free imaging technique, for diagnosing ectopic ureters (EUs) in dogs. Five dogs were presented for evaluation of recurrent urinary tract infections and urinary incontinence. All dogs were bright and alert on presentation, and physical examination findings, complete blood count, and serum biochemistry results were within reference intervals. Plain radiographs and conventional ultrasonography did not reveal any appreciable changes. Following aseptic urethral catheterization, CE‐VUS was performed by infusing a second‐generation microbubble‐based ultrasound contrast agent into the urinary bladder. The contrast agent was observed in both ureters and pelvicalyceal systems in cases one and five and in the left ureter and pelvicalyceal system in cases two, three, and four. Displacement of the ureteral orifices was documented caudal to the bladder trigone in all dogs with EUs. Additionally, a ureterocele associated with the left EU was identified in case three. These findings were consistent with vesicoureteral reflux secondary to EUs. The diagnosis was confirmed by radiographic cystography and/or computed tomography excretory urography (CTEU). CE‐VUS appears to be a useful, radiation‐free imaging technique for evaluating urinary tract morphology and function in dogs. To the authors’ knowledge, no previous studies have reported the clinical use of CE‐VUS for the diagnosis of EUs in dogs.

## 1 Introduction

Ureteral ectopia is the most common cause of continuous or intermittent urinary incontinence in young dogs and cats [1]. It is a congenital abnormality in which one or both ureteral orifices are located aberrantly distal to the bladder trigone, terminating in a position other than the normal one [1, 2, 3, 4]. Embryologically, ectopic ureters (EUs) are believed to develop from abnormal development of the ureteral bud because of its abnormal positioning along the mesonephric duct during embryogenesis [3, 4, 5, 6]. Abnormal growth and development of mesonephric and metanephric duct systems lead to abnormal ureteral tube termination [4, 5]. Common sites of ectopically displaced ureteral orifices include the bladder neck, urethra, vagina, vestibule, or uterus [3, 7, 8, 9].

A variety of anatomical configurations of EUs have been reported [3, 7]. EUs can be unilateral or bilateral and are classified as intramural or extramural [5, 8, 9]. In dogs, more than 95% of cases are intramural [2, 4]. Extramural EUs completely bypass the urinary bladder without anatomic attachment [3, 9], whereas intramural EUs course through the mucosal and submucosal layers of the bladder wall before exiting at an abnormal site [5, 8, 9, 10].

Female dogs are more frequently affected and often present with more severe urinary incontinence, likely due to their shorter urethral length [2, 3]. EUs are uncommonly reported in male dogs and may remain undiagnosed if urinary incontinence is absent, possibly because of the longer male urethra or presence of the prostate [10]. EUs are extremely rare in cats [4, 10, 11]. The condition occurs in both purebred and mixed breed dogs, with Labrador Retriever, Golden Retriever, Newfoundland, and Siberian Husky among the predisposed breeds [2, 3, 5, 7].

EUs are frequently associated with concurrent urinary tract anomalies, including renal agenesis or dysplasia, hydronephrosis, hydroureter, tortuosity, pelvic bladder, urachal remnants, and abnormal ureterovesical junction morphology [4, 5]. Because of this, the entire urinary system must be evaluated [3, 5].

Differential diagnoses should incorporate functional or structural anomalies of the lower urinary tract, and other neurogenic causes of incontinence [3, 4, 5]. Urinary tract infections (UTIs) are commonly associated with ectopia and have been reported in 64% of patients with EUs [3, 4, 12].

Diagnosis of EUs has traditionally been performed using contrast radiographic procedures, including intravenous excretory urography. However, these studies can correctly identify only approximately 78% of EUs [1, 3, 5], whereas more advanced diagnostic modalities such as computed tomographic excretory urography (CTEU) and cystoscopy improve accuracy but require general anesthesia [2, 4, 9]. A combination of these diagnostic procedures can be used to evaluate the path of the distal ureter, identify the ureteral orifice, and recognize associated abnormalities of the entire urinary tract [3, 4, 9, 12]. Contrast‐enhanced voiding urosonography (CE‐VUS) offers a dynamic and minimally invasive imaging alternative [13, 14].

The aim of the current study is to describe the application and preliminary diagnostic performance of CE‐VUS for the diagnosis of suspected ureteral ectopia in dogs, using contrast radiographic procedures or CTEU findings as the gold standard for comparison [15]. There are no published studies evaluating the diagnostic efficacy of CE‐VUS for the diagnosis of ureteral ectopia in dogs.

## 2 Materials and Methods

Medical records of five client‐owned dogs presented for investigation of urinary incontinence at the Companion Animal Clinic, Faculty of Veterinary Medicine, Aristotle University of Thessaloniki (AUTH), Thessaloniki, Greece, between 2018 and 2023, were reviewed retrospectively. The study was conducted in accordance with Hellenic laws on animal care and was approved by the institutional ethics committee. Written informed consent was obtained from all owners after providing detailed information about the purpose and the procedure of the study.

For inclusion in this study, dogs with suspected EU, based on clinical signs (urinary incontinence), underwent evaluation with CE‐VUS. The diagnosis was subsequently confirmed preoperatively by CTEU or excretory urography and intraoperatively confirmed as intramural or extramural EU during ventral cystotomy. Data collected included signalment, clinical signs, physical examination findings, hematologic findings, urinalysis, and procedural report. CE‐VUS examination was performed and assessed at the time of initial evaluation by two certified radiologists (CE‐VUS examination was performed and assessed at the time of initial evaluation by two veterinarians, PP and TG, having respectively 27 and 9 years of imaging experience) who also determined whether dogs met inclusion criteria. CTEU or excretory urography reports were generated by the radiology section of AUTH.

CE‐VUS examinations were performed in conscious or lightly sedated dogs, following previously described protocols [13, 14]. An ultrasound unit (MyLab X8 VET; ESAOTE SpA, Genova, Italy) equipped with a microconvex transducer mc 3–11 (3–11 MHz) and contrast‐tuned imaging (CnTI) software was used. CE‐VUS was performed immediately following B‐mode ultrasonography, with the same transducer switched to contrast mode. During CE‐VUS examination, the mechanical index (MI) was set from 0.08 to 0.10, and a single focal zone was used. Split‐screen display allowed simultaneous visualization of gray‐scale and contrast‐enhanced images. Two‐minute digital video clips were taken immediately after contrast infusion and stored in the hard disk of the ultrasound unit.

Scanning was performed under manual restraint with the dog in right lateral recumbency, according to the preference of the operator, after the insertion of a 20‐ or 22‐ga IV catheter (B/Braun Medical Inc, Hessen, Germany) in the cephalic vein to ensure safety. Mild sedation was provided in uncooperative or aggressive dogs using dexmedetomidine hydrochloride (Dexdomitor; Orion Corporation Espoo, Finland) at a dose of 200 µg/m^2^ intramuscularly. The urinary bladder was catheterized aseptically using a 6‐ to 10‐F Foley catheter (Jorgen Kruuse A/S, Langeskov, Denmark) and then completely emptied prior to contrast administration [13, 14].

All CE‐VUS examinations were performed by the same two operators (PP and TG). The ultrasound contrast agent (UCA) used was sulfur‐hexafluoride (SonoVue). After aseptic catheterization and bladder emptying, 1 mL of reconstituted UCA was injected into a bottle of 250 mL sterile normal saline (sodium chloride 0.9%; Demo SA, Athens, Greece) shortly before each examination, and the content was infused by gravity until the drop rate stopped or voiding started.

Transabdominal examination of both kidneys and midline prepubic approach for urinary bladder neck and proximal part of the urethra assessment was performed before and immediately after the onset of spontaneous micturition while withdrawing the catheter. All dogs urinated spontaneously when the catheter was removed, without the need to apply external pressure to the bladder. Removal of the catheter during voiding was essential for accurate assessment of urethral morphology and identification of orthotopic or ectopic ureterovesicular junctions. The bladder neck was carefully examined to identify ureterovesicular junctions and orifices. Additionally, identification of ureteral jets in the bladder trigone region was used to characterize the ureter as orthotopic. Both kidneys were assessed for microbubble reflux into the renal pelvis, indicating vesicoureteral reflux (VUR) may be associated with EU. Both the presence of VUR and the course of the ureter bypassing the normal ureteral opening into the bladder neck indicated an EU.

After CE‐VUS examination, each dog underwent excretory urography or CTEU to confirm the imaging findings. Subsequently, all dogs underwent surgery, and a midline cystotomy was performed to visually confirm the location and type of ureteral insertion (intramural or extramural).

## 3 Results

Five intact female dogs diagnosed with EUs were included in the study (Table 1). CE‐VUS examinations with SonoVue were successfully performed in all dogs presented for evaluation of intermittent (1/5) or persistent (4/5) urinary incontinence at a young age. All dogs (5/5) were confirmed to have EUs on the basis of the intraoperative findings. Of the 10 ureters evaluated, two dogs had bilateral EUs, and three dogs had unilateral EUs, yielding a total of seven ectopic and three normal ureters. The ureteral course of all EUs (5/5) was determined to be intramural. In all EUs, VUR and abnormal course were detected.

**TABLE 1:** Clinical findings and diagnostic imaging results in five dogs.

Case	1	2	3	4	5
Sex (male–female)	Female	Female	Female	Female	Female
Age (months)	12	4.5	7	11	8
Weight (kg)	4.6	5	6.6	9.7	12
Breed	Jack Russel Terrier	French Bulldog	Lhasa Apso	Mixed breed	Mixed breed
Castration status (intact—neutered)	Intact	Intact	Intact	Intact	Intact
Incontinence	Persistent	Intermittent	Persistent	Persistent	Persistent
Urinary tract infection (UTI)	No	No	Yes (*Escherichia coli* + *Klebsiella pneumoniae*)	No	No
Renal pelvis or/and ureteral dilation	No	No	Yes	No	No
Ectopia (unilateral or bilateral/intramural or extramural)	Bilateral/Intramural	Unilateral left ureter/intramural	Unilateral left ureter/intramural	Unilateral left ureter/intramural	Bilateral/Intramural
Concurrent abnormalities	No	No	Ureterocele	No	No

Median age at the time of diagnosis was 8.5 months (range, 4.5–12 months). The affected dogs represented various breeds, including two mixed breeds (2/5, 40%), and one each of Jack Russell Terrier, French Bulldog, and Lhasa Apso (1/5, 20%). UTI was detected by aerobic and anaerobic bacterial culture in one dog (20%) with EU. The remaining four dogs (80%) had negative urine cultures.

The assessment of the urethra during spontaneous micturition was successfully performed in all dogs (100%) in whom voiding was elicited during the examination. Free and continuous flow of the contrast through the urethra was observed in all animals. During CE‐VUS, an EU was identified as a tubular structure filled with contrast medium passing the urinary bladder wall caudally and entering the urethra lumen distal to the bladder trigone (Figure 1A–F). The presence of contrast medium in the EU was consistent with VUR resulting from an incompetent vesicourethral sphincter. In these cases, contrast medium was also detected in the renal pelvis. All ectopic ureteral openings were located within the proximal portion of the urethra. In one dog with EU, concurrent ureterocele was identified.

FIGURE 1(A–F) CE‐VUS images of the left ectopic ureter (white arrows) in a dog. Consecutive transverse planes from the mild level of the urinary bladder (A) to the proximal level of the urethra (F). Note the presence of contrast medium in the left ectopic ureter (arrow). This is consistent with vesicoureteral reflux. The left ureter bypasses the bladder and opens into the proximal portion of the urethra (arrowheads).

**Figure:**
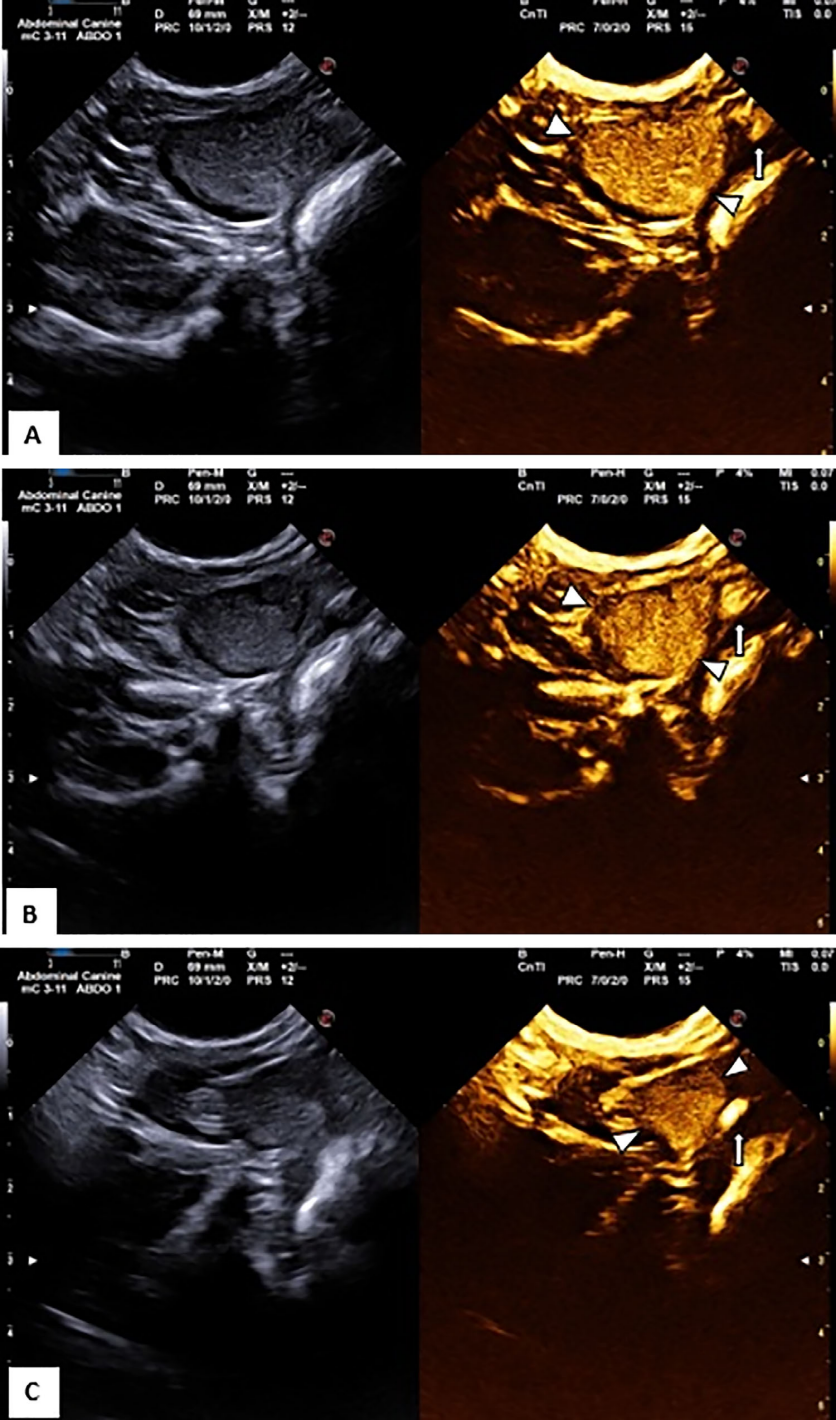

**Figure:**
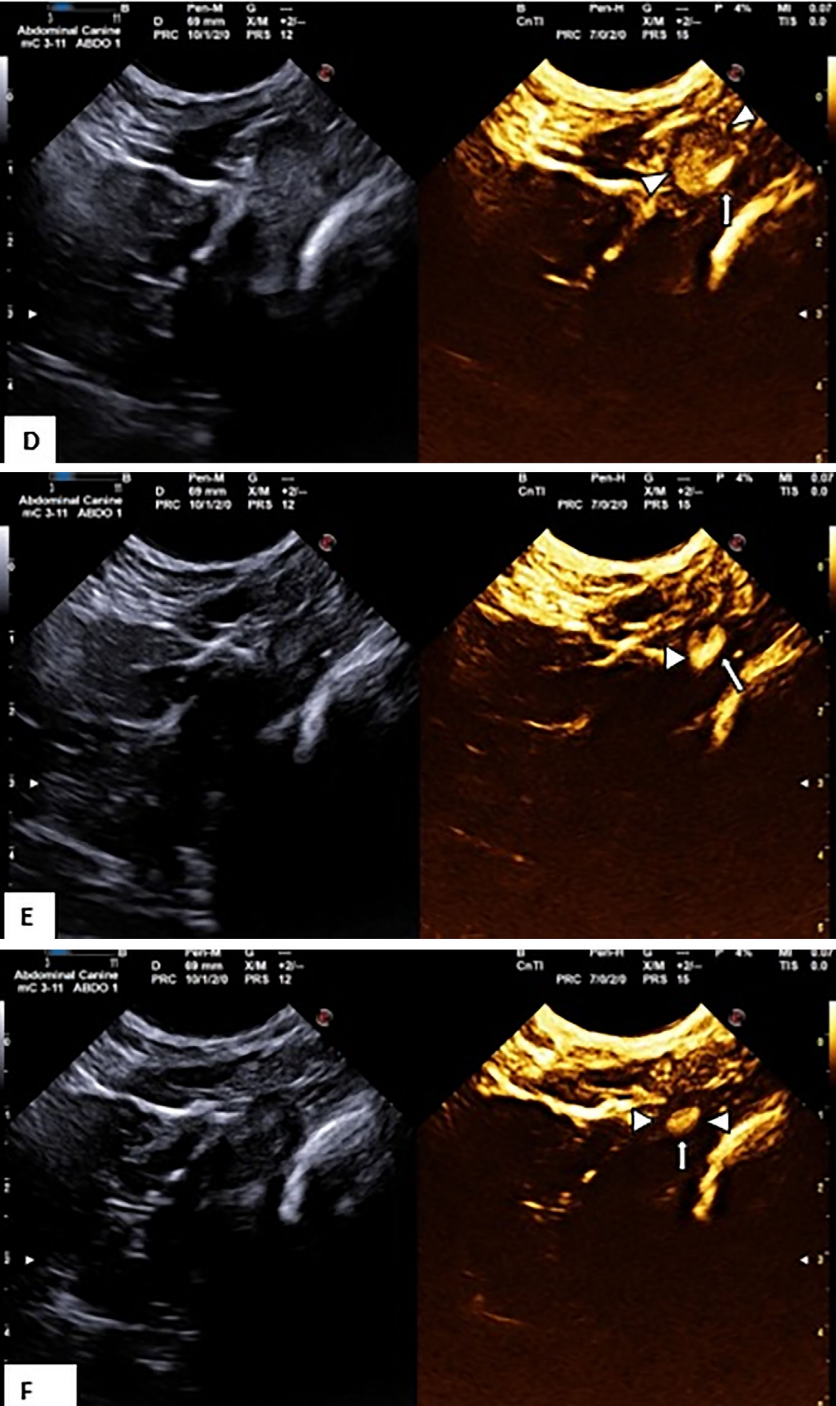

**FIGURE 2:**
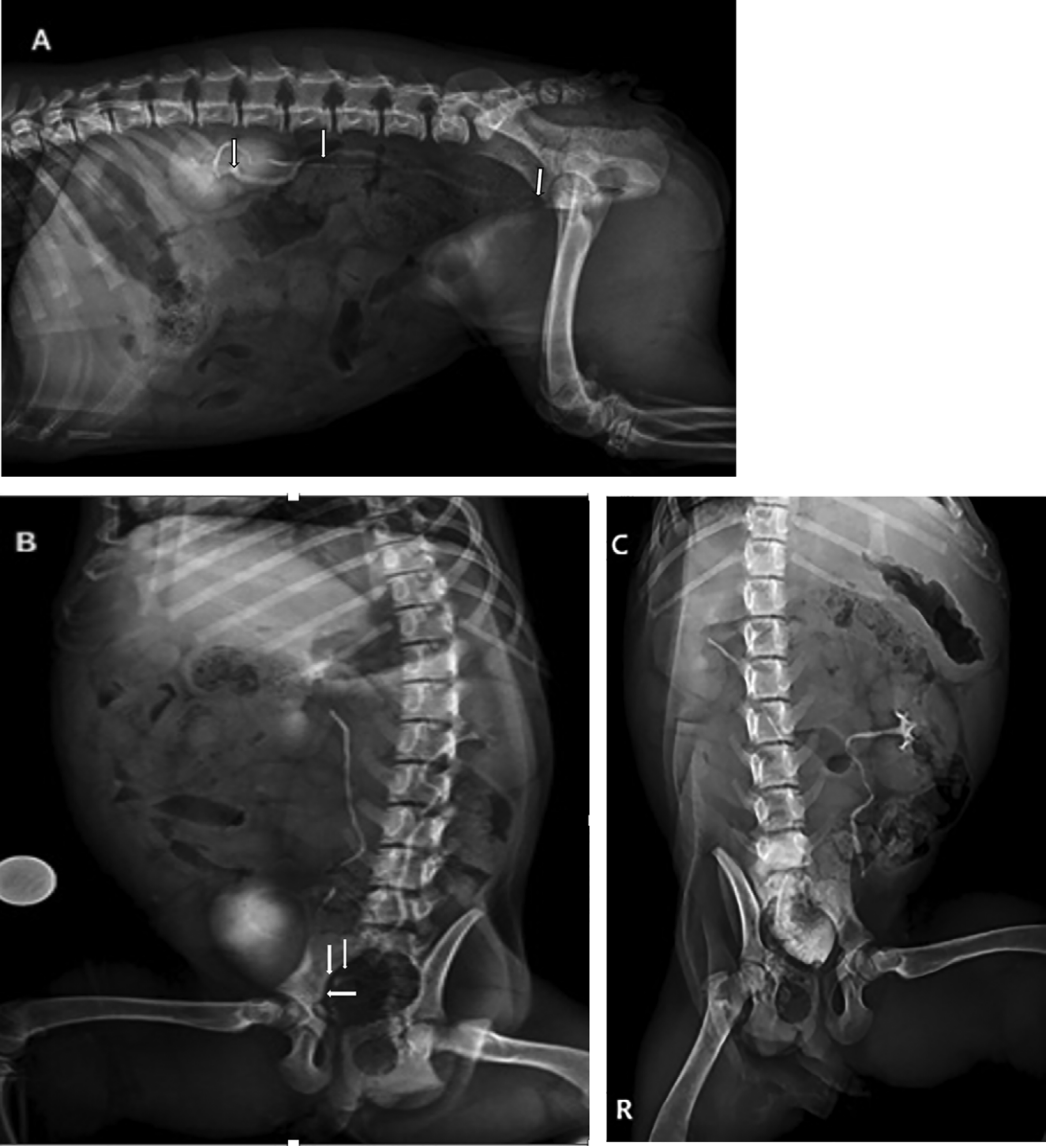
Radiographic contrast urography in the same dog (Figure 1). Abnormal ureteral tube termination into the urethra was observed in lateral (A), ventrodorsal (B), and oblique plane (C). Note the presence of vesicoureteral reflux in the orthotopic right ureter and in the ectopic left ureter (white arrows).

CE‐VUS findings were in accordance with those obtained by excretory urography or CTEU (Figure 2A–C). All results were confirmed intraoperatively during midline cystotomy, which allowed direct visualization of ureteral openings. The total procedure time for CE‐VUS ranged from 22 to 30 min (median, 26.2 min). No procedure‐related complications were reported.

## 4 Discussion

This study describes the first use of CE‐VUS for the diagnosis of EUs in dogs. In all five cases, CE‐VUS successfully identified intramural EUs, and the findings were corroborated by the other diagnostic imaging modalities and surgical exploration. These results highlight the feasibility and diagnostic potential of CE‐VUS as an alternative to traditional imaging techniques, a non‐ionizing, dynamic imaging modality for canine congenital urinary tract abnormalities.

In pediatric human medicine, CE‐VUS has become a validated, widely adopted alternative to voiding cystourethrography (VCUG) for evaluation of VUR and other abnormalities of the lower urinary tract, especially in children [16, 17, 18, 19, 20]. It has been widely accepted as a diagnostic method of VUR across Europe and the United States because of its high sensitivity and specificity, real‐time dynamic assessment of ureteral jets, and the absence of ionizing radiation [16, 17, 18]. In veterinary medicine, reports of CE‐VUS are limited but promising. There are three studies investigating the urinary bladder and urethra in dogs and VUR in young dogs with the use of CE‐VUS examination [13, 14, 21].

The diagnosis of EUs in dogs remains challenging. Conventional diagnostic techniques such as plain and contrast radiographic techniques, including radiographic excretory urography, positive‐contrast cystography, double‐contrast cystography, retrograde urethrography, vaginocystography, and fluoroscopy, have long been used, but their sensitivity is limited [1, 6]. Excretory urography has long been the method of choice in diagnosing EUs. Contrast radiography allows correct localization of ureteral termination in only 62%–77% of cases [3, 6]. Radiographic techniques are further impeded by superimposition artifacts and poor ability to differentiate intramural from extramural courses [3, 10]. Additionally, to perform classic urethrography procedures (such as radiographic and CT procedures), sedation or general anesthesia is required, and this is the main limitation of these examinations, especially in critically ill patients. On the contrary, CE‐VUS examination may be performed without the need for sedation or general anesthesia [13, 16, 19].

Retrograde urethrography has limited value because of the presence of the catheter within the urethral lumen, which may obscure or obstruct a displaced ureteral orifice [6]. However, retrograde vaginocystography is performed without a catheter in the urethral lumen and has been shown to be very useful in locating and evaluating the terminal orifice of the EU, the entire length of the urethra, and the vaginal contour [3, 6]. In our study, the catheter withdraws into the distal urethra during dog micturition, allowing further proximal urethra morphology and anatomy assessment. This maneuver is very important to visualize the ureteral openings into the proximal portion of the urethra and the EU course.

Ultrasonography, traditionally considered of limited value, has been shown to achieve high sensitivity and specificity when performed by experienced operators or when combined with UCAs [2, 19, 21, 22]. Although there are certain technical difficulties, ultrasonography has comparable accuracy to contrast radiography, but it takes less time and avoids the need for multiple radiographs or image intensification to identify the termination of the ureters. Ureterovesicular junctions in many dogs are very small structures that are difficult to visualize during ultrasound. However, the expected origin of a ureteral jet may contribute to identifying the ureteral opening site. Use of color‐flow Doppler ultrasound facilitates the recognition of ureterovesicular junctions [13, 22].

CTEU is now widely considered the imaging modality of choice, with reported sensitivity up to 91% for detection of EUs in dogs [5, 9]. CTEU offers high‐resolution multiplanar reconstructions, avoids superimposition, and allows assessment of concurrent abnormalities such as ureteral dilation, tortuosity, and ureteroceles [5, 9, 12]. However, it requires general anesthesia, specialized equipment, and exposes patients to ionizing radiation [5, 9].

Cystoscopy remains the reference standard for diagnosis, allowing direct visualization of ureteral orifices and simultaneous therapeutic intervention [1, 23, 24]. Cystoscopy enables precise differentiation between intramural and extramural ectopia and is the only modality that reliably detects subtle intramural courses [6, 24]. Nevertheless, cystoscopy requires specialized training, expensive instrumentation, and general anesthesia, limiting its availability in general practice [1, 24].

Detection of concurrent abnormalities is clinically important. Ureteroceles, hydroureter, and hydronephrosis are detected in up to 70%–94% of dogs with EUs [4, 6]. In our study, CE‐VUS identified a ureterocele, reinforcing its capacity to diagnose not only primary ectopia but also associated abnormalities. Ureteroceles can cause obstruction, recurrent UTIs, and renal impairment, necessitating accurate preoperative identification [12, 25]. Complex anomalies, such as ureteroceles, duplications, and agenesis, may have a great impact on prognosis and surgical planning [5, 11, 26]. CE‐VUS, by enabling dynamic functional assessment, is particularly suited to detect these abnormalities [16, 19].

In this study, CE‐VUS examination was performed first and then either CTEU or radiographic contrast urography. This was done because we did not want to be biased by knowing the results from the other imaging techniques. Additionally, this was according to the latest data published from studies performed in the same manner as in our study [13, 17, 20, 27, 28].

This pilot study demonstrates proof‐of‐concept that CE‐VUS is a feasible and diagnostically valuable imaging modality in dogs with EUs. Strengths include correlation with reference‐standard imaging and surgical findings, and dynamic functional assessment not possible with static techniques. The ability to perform CE‐VUS without exposing both the patient and the examiner to ionizing radiation, the dynamic character of the examination, and the high safety of the procedure appear to be the essential advantages of this modality.

The present study is limited by the small number of dogs included, reflecting the rarity of diagnosed EUs assessed at a single referral center. Furthermore, patient cooperation may be a limitation in veterinary practice, although sedation is not always required. The lack of male dogs is a limitation in our pilot study. Larger prospective studies are required to validate diagnostic accuracy, reproducibility, and cost‐effectiveness of this method compared to other imaging modalities.

In conclusion, CE‐VUS appears to be a promising, noninvasive diagnostic modality for detecting EUs in dogs. It combines the advantages of non‐ionizing radiation, dynamic functional assessment, and feasibility in conscious or lightly sedated patients. Our findings suggest that CE‐VUS could complement or even replace more invasive modalities, such as CTEU, in the diagnostic work‐up of urinary incontinence in dogs. With further validation, CE‐VUS has the potential to expand access to accurate, safe, and cost‐effective diagnosis of congenital lower urinary tract anomalies in veterinary medicine on a routine basis.

## Author Contributions

**Georgia Trikoupi**: investigation, writing – original draft, methodology, writing – review and editing, conceptualization, validation. **Lysimachos Papazoglou**: supervision, writing – review and editing. **Paraskevi Papadopoulou**: conceptualization, investigation, writing – review and editing, supervision, validation, methodology.

## Disclosure

This manuscript has not been published previously and has not been presented, in whole or in part, at any scientific meeting or conference.

## Ethics Statement

All procedures were conducted in accordance with national legislation on animal care and approved by the institutional ethics committee.

## Consent

Written informed consent was obtained from all dog owners prior to participation.

## Conflicts of Interest

The authors declare no conflicts of interest.

## Data Availability

The data supporting the findings of this study are available from the corresponding author upon reasonable request.
